# COVID-19–Related Misinformation among Parents of Patients with Pediatric Cancer

**DOI:** 10.3201/eid2702.203285

**Published:** 2021-02

**Authors:** Jeanine P.D. Guidry, Carrie A. Miller, Albert J. Ksinan, Jennifer M. Rohan, Marcia A. Winter, Kellie E. Carlyle, Bernard F. Fuemmeler

**Affiliations:** Virginia Commonwealth University, Richmond, VA, USA

**Keywords:** respiratory infections, severe acute respiratory syndrome coronavirus 2, SARS-CoV-2, SARS, 2019 coronavirus disease, viruses, coronavirus, pediatric cancer, misinformation

## Abstract

We conducted a survey among 735 parents to determine differences in endorsement of misinformation related to the coronavirus disease pandemic between parents of children in cancer treatment and those with children who had no cancer history. Parents of children with cancer were more likely to believe misinformation than parents of children without cancer.

Medical misinformation and unverifiable content about the coronavirus disease (COVID-19) pandemic have been propagated at an alarming rate, particularly on social media (*1*). Such misinformation may confer increased risk for nonadherence with COVID-19–related guidelines as well as ongoing medical regimens (*2*,*3*), which is particularly concerning for patients who are immunocompromised, such as children with cancer (*4*). The extent to which COVID-19 misinformation is believed by parents is not yet known, nor is it known whether parents of medically vulnerable children are more or less susceptible to misinformation than parents of children who are not medically vulnerable. Although parents of children with cancer may be more attentive to online medical information, rendering them more susceptible to misinformation, they may also be more discerning in what they endorse. We sought to determine whether parents of children with cancer are more or less vulnerable to COVID-19–related misinformation than their counterparts who have generally healthy children.

The panel survey firm Qualtrics (https://www.qualtrics.com) conducted a survey among 735 parents of children 2–17 years of age (n = 315 currently in cancer treatment, 38.7% female parent/caregiver; n = 420 without a cancer history, 67.1% female parent/caregiver) during May 1–31, 2020. Participants were asked to endorse a series of COVID-19–related misinformation statements taken from the World Health Organization’s website, with the following scale: “Definitely untrue,” “Likely untrue,” “Not sure if untrue/true,” “Likely true,” and “Definitely true” (Figure) (*5*). Participants also answered questions about their highest attained education (dichotomized: college degree or less than college degree), sex, age, and race (dichotomized: white and nonwhite); an item also asked participants how much stress the COVID-19 pandemic has caused them, rated on a scale from 1 = “Not at all stressed” to 5 = “Extremely stressed.”

**Figure:**
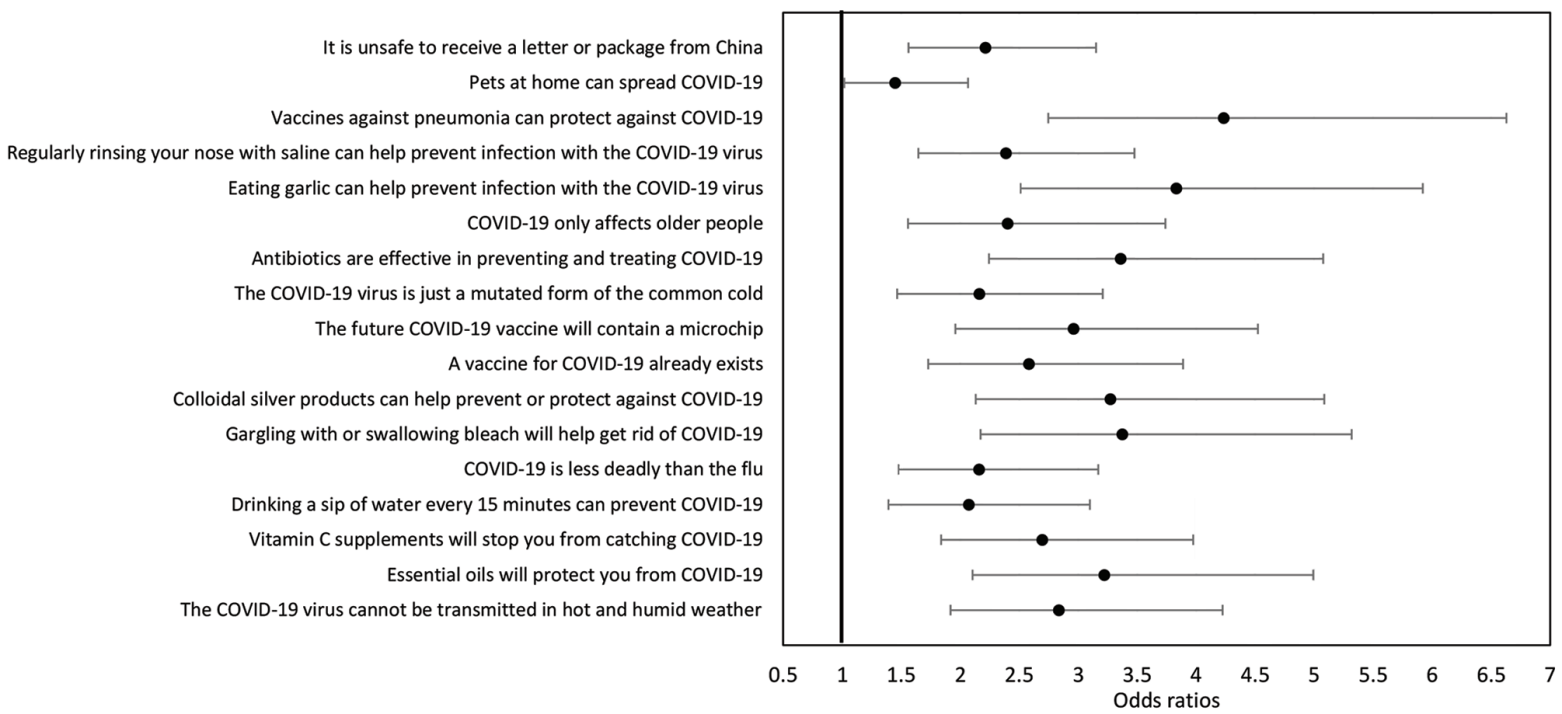
Forest plot of odds ratios for parents of children with cancer (as opposed to parents of children without cancer) predicting each dichotomized COVID-19 misinformation item (“definitely true” and “likely true” answers coded as 1, others as 0). Results are adjusted for sex, age, race, and education of parent as well as COVID-19–related stress. COVID-19, coronavirus disease.

First, we evaluated the fit of a single-factor confirmatory factor analysis model with misinformation items as indicators. The fit was adequate: χ^2^ (118) = 424.90, p<0.001, comparative fit index (CFI) = 0.94, root mean square error of approximation = 0.07. The reliability of the scale was α = 0.94. Next, we used the confirmatory factor analysis model as a dependent variable in a structural equation model, with parental age, sex, race, education, perceived stress from COVID-19, and parent group as predictors (Table). The fit was adequate: χ^2^ (198) = 608.60, p < 0.001, CFI = 0.93, root mean square error of approximation = 0.06. Parents of children with cancer were more likely to believe misinformation compared with parents of children without cancer. Believing misinformation was also more likely for fathers, younger parents, and parents with higher perceived stress from COVID-19. As a follow-up to this summative analysis, we evaluated each of the misconception items separately to determine the likelihood of endorsement of each item among parents of children with cancer compared with their counterparts using a logistic regression analysis (dichotomizing each item as definitely true and likely true = 1, others = 0) controlling for age, race, education, sex, and perceived stress (Figure). 

**Table:** Results from structural model predicting belief in COVID-19 misinformation among parents of children with and without pediatric cancer*

Characteristic	B	SE	p value	β
Male	0.18	0.05	<0.001	0.16
Age	−0.01	0.01	<0.001	−0.16
Nonwhite	−0.07	0.04	0.169	−0.05
College degree	−0.01	0.05	0.725	−0.01
COVID-19 stress	0.06	0.02	0.001	0.12
Parent of patient with pediatric cancer	0.37	0.06	<0.001	0.33

*B, unstandardized beta; COVID-19, coronavirus disease.

This study’s main finding was that parents of children with cancer were more likely to endorse misinformation about COVID-19, as well as more likely to believe myths associated with COVID-19 prevention as opposed to those related to COVID-19 susceptibility (Appendix). It is not completely clear why parents of children with cancer are more vulnerable to misinformation. Parents of children with cancer may be at greater risk of exposure to misinformation as a result of greater levels of COVID-19–related stress, resulting in more time spent looking for information online. Moreover, the increased stress levels reported by these parents could be affecting their information-processing abilities, making them more likely to use heuristics or cues rather than more critical, central processing routes of assessing information credibility (*6*).

From the perspective of health behavior theory, parents who feel high levels of fear should be most likely to seek out efficacious responses to ease their fears (*7*). This tendency could offer one explanation for why prevention-focused myths were more likely to be endorsed by parents of patients with pediatric cancer.

The mortality rate for pediatric cancer has increased during the COVID-19 pandemic as a result of delayed access to medical care; misinformation related to COVID-19 may also be a contributing factor (*8*). Although this study was focused on parents of children with cancer, it is possible that parents of children with other chronic diseases, as well as adult patients and caregivers, may experience similar patterns. Future studies should investigate the extent in which these findings hold in similar high-risk populations.

This study’s results suggest that healthcare professionals working in pediatric oncology, in particular, should be aware of the potentially high endorsement of COVID-19 misinformation among parents of their patients across the illness trajectory, from new diagnosis to survivorship, and should proactively address this in routine visits as well as tailored written materials. The evolving nature of our understanding of COVID-19 necessitates coordinated and diligent efforts to reduce illness and death. Paramount among these efforts is the development of innovative preventive interventions to combat COVID-19–related misinformation.

Appendix Additional information on study of COVID-19 misinformation among parents of patients with pediatric cancer.
