# Supplementary material for: COVID-19–Related Misinformation among Parents of Patients with Pediatric Cancer
Source: Emerg Infect Dis. 2021 Feb;27(2):650–2. doi: 10.3201/eid2702.203285 (PMC7853539; doi:10.3201/eid2702.203285)
Supplement: Appendix — Additional information on study of COVID-19 misinformation among parents of patients with pediatric cancer. [file 20-3285-Techapp-s1.pdf]

Article DOI: https://doi.org/10.3201/eid2702.203285

COVID-19–Related Misinformation among Parents of Patients with Pediatric Cancer

Appendix

The 2019 novel coronavirus disease (COVID-19) has spread quickly throughout the world, with 50,676,072 confirmed cases and 1,261,075 confirmed deaths as of November 10, 2020 (*1*). The viral outbreak has been accompanied by an infodemic—a surfeit of information spread widely and rapidly—including what United Nations Secretary General António Guterres has called a "pandemic of misinformation" (*2*,*3*). The misinformation content related to COVID-19 consists of a stream of conspiracy theories, unverifiable content, and rumors about the outcomes, prevention, and cures of the disease (*4*,*5*). Much of the COVID-19 misinformation has been disseminated on social media (*4*,*6*,*7*). For example, studies examining COVID-19 related content on Twitter show an alarming level of misinformation spread (*2*,*6*,*7*). That COVID-19 misinformation has been identified across numerous social media platforms (*3*) is not particularly surprising, given that health misinformation, as compared with accurate, reliable information, is more likely to be shared on social media platforms than via other communication channels (*8*).

The widespread proliferation of COVID-19 misinformation is concerning for several reasons. First, once misinformation is believed as factual, these false beliefs can influence behavior, reasoning, and memory, and be very difficult to correct (*9*,*10*). Second, among the general population, misinformation can hinder the practice of protective behaviors that can be effective at slowing or decreasing the spread of the virus (*3*). Third, among persons with chronic diseases and cancer, misinformation is especially problematic because it may disrupt adherence to ongoing treatment regimens (*11*,*12*) or result in skipped or delayed medical care, thereby interfering with optimal treatment outcomes (*13*). Knowing the extent to which misinformation around COVID-19 is taking hold among persons with chronic disease or cancer patients and their families is key to discern because addressing it could help improve health outcomes among this vulnerable group of persons during a pandemic such as we have now.

Misinformation may be particularly problematic among parents of children with cancer. To make the best decisions for the care of their children, these parents must be able to understand complex medical information; discern how to keep their children free from disease, especially if they are immunocompromised; and help them adjust to the demands of treatment and recovery. On one hand, parents of pediatric cancer patients, who generally have more experience with medical information and the healthcare system, may be more discerning about COVID-19–related information than their counterparts who have generally healthy children. On the other hand, the COVID-19 epidemic may increase anxiety and fear among parents of children with cancer. These parents may be more attentive to online medical information; thus, they may have greater exposure to misinformation. Parents of children with cancer are also likely to be active on social networking sites (e.g., Facebook groups) relevant to their child's health condition (*14*) and, thus, could be exposed to misinformation posted by other members.

No research to date has investigated COVID-19 misinformation beliefs among parents of patients with pediatric cancer or whether parents of medically vulnerable children are more or less susceptible to misinformation than parents of non-medically vulnerable children. Further consideration is warranted to investigate the potential for parents of children with cancer to be more susceptible to misinformation because endorsement of misinformation could potentially disrupt their children's care or result in poorer health outcomes among their children. The purpose of this study was to determine whether parents of children with cancer are more or less vulnerable to COVID-19–related misinformation than their counterparts who have generally healthy children.

Methods

Sample

Wes used the panel survey firm Qualtrics to administer an online survey. Data were collected from 735 parents of children 2–17 years of age during May 1–31, 2020. Quota sampling was used to ensure that about half the sample would consist of parents with children currently undergoing cancer treatment and one-half of parents of children without a history of cancer. The study was approved by the Institutional Review Board of Virginia Commonwealth University.

Measures

Parent Demographics

Participants self-reported their gender ("male [1]," with "female [0]" as a reference group), age in years, and race. Race was dichotomized into "white (0)" or "non-white (1)." Participants also indicated their highest education level attained. This was dichotomized into either "college degree (1)" or "less than college degree (0)." Parents were assigned a label according to their child's cancer status ("Pediatric cancer parent [1]," or "non-pediatric cancer parent [0])."

COVID-19 Stress

A single item, "How much stress has the coronavirus disease (COVID-19) caused you?," was used to question how much stress the COVID-19 pandemic had caused participants. Responses were provided on a 6-point scale from "not at all stressed (0)" to "extremely stressed (5)."

COVID-19 Misinformation

Participants were asked to endorse a series of 17 COVID-19–related misinformation statements taken from the World Health Organization's website, using the following 5-point response scale: "definitely untrue," "likely untrue," "not sure if untrue/true," "likely true," and "definitely true." The items included statements related to susceptibility to (e.g., "COVID-19 only affects older people," "The COVID-19 virus cannot be transmitted in hot and humid weather") and prevention of COVID-19 (e.g., "Eating garlic can help prevent infection with the COVID-19 virus," "Gargling with or swallowing bleach will help get rid of COVID-19"). A complete description of misinformation items is provided in Appendix Table 1 (*15*).

Plan of Analysis

In the first step, we computed descriptive statistics of the study variables and compared them across our study groups. Then we tested whether the misinformation items might be subsumed under a single construct. For this, we estimated a single-factor confirmatory factor analysis (CFA) model with items as indicators. Then, the single-factor model for misinformation was included as a dependent variable in a full structural model, with gender, age, race, education level, stress from COVID, and parent group entered as predictors. Finally, we assessed the model

separately with each of the misinformation items dichotomized (“definitely true” and “likely true” [1], or other [0]) in a series of logistic regression analyses, including the aforementioned covariates. All analyses were performed in R 4.0.2 (www.r-project.org). The structural equation modeling (SEM) was done using the lavaan package and MLRestimator with robust standard errors to deal with nonnormal distribution of the data. Logistic regressions were estimated using the R statistics package.

Results

Of this sample, 42.9% (n = 315) consisted of parents of children currently in cancer treatment, with 38.7% being female parent/caregiver, and 57.1% (n = 420) of parents of children without a cancer history, with 67.1% female parent/caregiver (Appendix Table 2). Study results showed significant differences between the study groups for all the covariates. There were slightly more females among the parents in the non-cancer group (67% versus 61%). The parents in the pediatric cancer group showed higher educational attainment, as 81% had at least a bachelor’s degree (compared to 50% in the non-cancer group). There were more white participants in the non-cancer group (88% versus 79%). The parents of pediatric cancer patients were younger (mean age = 36.5 [SD 7.9] versus 42.1 [SD 11.5]) and reported higher levels of stress due to COVID-19 (average score = 3.70 (SD 1.1) versus 3.13 (SD 1.3). In the next step, we compared mean levels of the COVID-19 misinformation items across the 2 groups (Appendix Table 1). The results indicate that across all items, parents of pediatric cancer patients reported higher endorsement of the misinformation items compared with parents of children with no cancer history.

In the next step, we estimated the 1-factor CFA model with misinformation items as indicators. Based on modification indices, we also added covariance for residuals between items “Vitamin C supplements will stop you from catching COVID-19” and “Essential oils will protect you from COVID-19.” The fit of the model was adequate: χ^2 (118) = 424.90, $p < 0.001$, CFI = 0.94, RMSEA = 0.07. The reliability of the scale was $\alpha = 0.94$.

Next, we extended the CFA model to a full structural model, with gender, age, race, education level, stress from COVID-19, and parent group entered as predictors. The results from this analysis are shown in Table 1 of the main article. The model showed higher endorsement of

misinformation among fathers compared with mothers ($\beta = 0.16$, $p<0.001$), younger parents ($\beta = -0.16$, $p<0.001$), and parents who reported more stress because of COVID-19 ($\beta = 0.12$, $p = 0.001$). No significant differences were found based on race or education attainment. The parents of children with cancer were significantly more likely to believe COVID-19–related misinformation then parents of children without cancer ($\beta = 0.33$, $p<0.001$).

Finally, we tested the misinformation items separately to assess whether the effect of parent group was present for all items or whether it was driven by only some of the items. The items were dichotomized and the results from logistic regressions are given in Appendix Table 3. They showed that pediatric cancer parent status significantly predicted each individual item above and beyond the covariates such that parents of children with cancer reported significantly higher levels of endorsing COVID-19 misinformation. The odds ratios for the group status are presented in the Figure in the main article.

Discussion

In this study, our primary aim was to determine to what degree parents of children with cancer may be more vulnerable to misinformation relative to their counterparts of parents with generally healthy children. Using a national panel survey methodology, we asked both parents of healthy children and parents of children who were in active treatment for cancer whether they believed or disbelieved a series of mostly false claims about COVID-19. Our main finding was that parents of children with cancer were more likely to endorse or believe false statements about the COVID-19 pandemic. That is, they were more vulnerable than parents of healthy children to misinformation. Other factors related to belief in misinformation were gender; with men more likely to believe misinformation; age, with younger parents more likely to believe misinformation; and reported level of stress as a result of the COVID-19 pandemic, with parents experiencing higher stress levels more likely to believe misinformation.

Across the 17 "myths," parents of children with cancer were more likely to believe myths associated with the prevention of COVID-19 (with odds ratios >3; e.g., "Vaccines against pneumonia can protect against COVID-19," "Eating garlic can help prevent infection with the COVID-19 virus," and "Antibiotics are effective in preventing and treating COVID-19", as opposed to myths related to the susceptibility to COVID-19 (with odds ratios <3; e.g., "COVID-

19 only affects older people” and “The COVID-19 virus cannot be transmitted in hot and humid weather”) compared with parents of generally healthy children (Figure). From the perspective of health behavior theory, parents who feel high levels of fear should be most likely to seek out efficacious responses to ease their fears (*16*). This could offer one explanation for why the prevention or solution-focused myths were more likely to be endorsed by parents of pediatric cancer patients. Although this relationship is typically predicted in an environment with accurate prevention information, the rapidly changing COVID-19 information environment may contribute to lowered abilities to identify efficacious responses to those characterized by misinformation.

It is not completely clear why parents of children with cancer are more vulnerable to misinformation. Parents of children with cancer may be at greater risk of exposure to misinformation as a result of greater levels of COVID-19–related stress, resulting in more time spent looking for information online. Moreover, the increased stress levels reported by these parents could be affecting their information-processing abilities, making them more likely to use heuristics or cues rather than more critical, central processing routes of assessing information credibility (*17*).

Being male was related to higher endorsement of misinformation. Studies show that, in general, mothers tend to be more involved in the care of chronically ill children than fathers (*18*). Perhaps this greater level of involvement with the medical care of the child gives women the opportunity to increase their health literacy skills, making them less vulnerable to misinformation. In addition, younger parents were more likely to endorse misinformation. Although it is unclear why this relationship exists, other studies of COVID-19 misinformation found similar results, with those endorsing conspiracy beliefs more likely to be younger (*19*). Allington and colleagues posited that the relationship might be explained by the heavier use of social media among younger populations, without the countereffect of information contained in broadcast media sources that tend to be used more by older adults (*19*). It is clear, though, that we need to know more about why parents of children with cancer are more likely to believe misinformation and additional research is needed to help address this. Recognizing the factors that relate to a higher endorsement of misinformation could inform communication strategies to reduce beliefs in false information.

This study should be viewed within the context of its strengths and limitations. One of its main strengths is the relatively large national sample size of parents of both healthy children and those in cancer treatment. Although this study was focused on parents of children with cancer, it is possible that parents of children with other chronic diseases, such as diabetes or asthma, or adult patients and caregivers may experience similar patterns such as those we see here. Future studies should investigate the extent to which these findings hold in similar high-risk populations.

Despite the large sample size, race still needed to be dichotomized into white and nonwhite categories for comparison, limiting the specificity of results for distinct racial minority groups. The constantly changing information landscape surrounding COVID-19 also presents a limitation, as the science of what is effective prevention and public health communications around recommended actions continually evolves with the rapidly advancing scientific understanding of COVID-19.

Understanding the full effect of the novel coronavirus is imperative, especially as it relates to vulnerable pediatric patient populations. This study is, to our knowledge, the first to explore COVID-19 misinformation among of parents of pediatric cancer patients. Parental beliefs in misinformation related to COVID-19 could have especially dangerous consequences for children with chronic illnesses or cancer because these patients are at increased risk for severe COVID-19–related complications (*20*). Within our sample, parents of children with cancer were significantly more likely than parents of children without a cancer history to endorse each of the 17 COVID-19 misinformation items.

Recent research has documented the toll of the COVID-19 pandemic on pediatric patients with chronic illnesses, specifically those with cancer (*13*,*21*). In fact, there is an increased incidence of delayed cancer diagnoses and high mortality rates in pediatric patients, some of which may be a result of the pandemic (*22*). Several parents are choosing to not seek medical care in fear of contracting COVID-19 and/or not having access to medical care because of pandemic-related healthcare office closures. The mortality rate for pediatric cancer has subsequently increased as a result of delayed access to medical care; misinformation related to COVID-19 may be a contributing factor in this (*22*). These results suggest that healthcare professionals working in pediatric oncology, in particular, should be aware of the potentially

high endorsement of COVID-19 misinformation among parents of pediatric cancer patients across the illness trajectory from new diagnosis to survivorship. Providers should both identify the presence in misinformation beliefs and proactively address COVID-19 misinformation in routine visits with pediatric oncology patients using specific, tailored written materials that complement verbal discussions. Providers should also use strategies such as “teaching back” methods to ensure that information is accurate, particularly because parents of children with cancer who feel most confused and most in charge of medical information and decision making may also feel less confidence and trust in their children’s medical doctors (*23*).

The evolving nature of our understanding of COVID-19 necessitates coordinated and diligent efforts to reduce illness and death. Paramount among these efforts is the development of innovative preventive interventions to combat COVID-19 related misinformation (*24*). Public health communicators must also endeavor to provide clear and consistent communications related to COVID-19.

References

1. World Health Organization. WHO coronavirus disease (COVID-19) dashboard. World Health Organization 2020 [cited 2020 Nov 10]. https://covid19.who.int

2. Pazzanese C. Battling the “pandemic of misinformation.” The Harvard Gazette. 2020 [cited 2020 Sep 10]. https://news.harvard.edu/gazette/story/2020/05/social-media-used-to-spread-create-covid-19-falsehoods

3. Tasnim S, Hossain MM, Mazumder H. Impact of rumors and misinformation on COVID-19 in social media. J Prev Med Public Health. 2020;53:171–4. PubMed https://doi.org/10.3961/jpmph.20.094

4. Depoux A, Martin S, Karafillakis E, Preet R, Wilder-Smith A, Larson H. The pandemic of social media panic travels faster than the COVID-19 outbreak. J Travel Med. 2020 Mar 3 [Epub ahead of print]. https://doi.org/10.1093/jtm/taaa031

5. Brennen JS, Simon F, Howard PN, Nielsen RK. Types, sources, and claims of Covid-19 misinformation. Reuters Institute 2020 Mar 3 [cited 2020 Sep 10]. https://reutersinstitute.politics.ox.ac.uk/types-sources-and-claims-covid-19-misinformation

6. Ahmed W, Vidal-Alaball J, Downing J, López Seguí F. COVID-19 and the 5G conspiracy theory: social network analysis of Twitter data. J Med Internet Res. 2020;22:e19458. PubMed https://doi.org/10.2196/19458

7. Kouzy R, Abi Jaoude J, Kraitem A, El Alam MB, Karam B, Adib E, et al. Coronavirus goes viral: quantifying the COVID-19 misinformation epidemic on Twitter. Cureus. 2020;12:e7255. PubMed https://doi.org10.7759/cureus.7255

8. Swire-Thompson B, Lazer D. Public health and online misinformation: challenges and recommendations. Annu Rev Public Health. 2020;41:433–51. PubMed https://doi.org/10.1146/annurev-publhealth-040119-094127

9. Ecker UK, Swire B, Lewandowsky S. Correcting misinformation—a challenge for education and cognitive science. In: Processing inaccurate information: theoretical and applied perspectives from cognitive science and the educational sciences. Rapp DN, Braasch JLG, editors. Cambridge (MA): MIT Press, 2014. p. 13–38.

10. Lewandowsky S, Ecker UK, Seifert CM, Schwarz N, Cook J. Misinformation and its correction: continued influence and successful debiasing. Psychol Sci Public Interest. 2012;13:106–31. PubMed https://doi.org/10.1177/1529100612451018

11. Clarke S-A, Eiser C. Health behaviours in childhood cancer survivors: a systematic review. Eur J Cancer. 2007;43:1373–84. PubMed https://doi.org/10.1016/j.ejca.2007.03.002

12. Casanova M, Pagani Bagliacca E, Silva M, Patriarca C, Veneroni L, Clerici CA, et al. How young patients with cancer perceive the COVID-19 (coronavirus) epidemic in Milan, Italy: is there room for other fears? Pediatr Blood Cancer. 2020;67:e28318. PubMed https://doi.org/10.1002/pbc.28318

13. Balduzzi A, Brivio E, Rovelli A, Rizzari C, Gasperini S, Melzi ML, et al. Lessons after the early management of the COVID-19 outbreak in a pediatric transplant and hemato-oncology center embedded within a COVID-19 dedicated hospital in Lombardia, Italy. Estote parati. Bone Marrow Transplant. 2020;55:1900–5. PubMed https://doi.org/10.1038/s41409-020-0895-4

14. Nagelhout ES, Linder LA, Austin T, Parsons BG, Scott B, Gardner E, et al. Social media use among parents and caregivers of children with cancer. J Pediatr Oncol Nurs. 2018;35:399–405. PubMed https://doi.org/10.1177/1043454218795091

15. World Health Organization. Coronavirus disease (COVID-19) advice for the public: myth busters. 2020 [cited 2020 Feb 29]. https://www.who.int/emergencies/diseases/novel-coronavirus-2019/advice-for-public/myth-busters

16. Witte K. Putting the fear back into fear appeals: the extended parallel process model. Commun Monogr. 1992;59:329–49. https://doi.org/10.1080/03637759209376276

17. Cameron KA. A practitioner's guide to persuasion: an overview of 15 selected persuasion theories, models and frameworks. Patient Educ Couns. 2009;74:309–17. PubMed https://doi.org/10.1016/j.pec.2008.12.003

18. Katz S. Gender differences in adapting to a child's chronic illness: a causal model. J Pediatr Nurs. 2002;17:257–69. PubMed https://doi.org/10.1053/jpdn.2002.126709

19. Allington D, Duffy B, Wessely S, Dhavan N, Rubin J. Health-protective behaviour, social media usage and conspiracy belief during the COVID-19 public health emergency. Psychol Med. 2020 Jun 9 [Epub ahead of print]. PubMed https://doi.org/10.1017/S003329172000224X

20. Sullivan M, Bouffet E, Rodriguez-Galindo C, Luna-Fineman S, Khan MS, Kearns P, et al. The COVID-19 pandemic: a rapid global response for children with cancer from SIOP, COG, SIOP-E, SIOP-PODC, IPSO, PROS, CCI, and St Jude Global. Pediatr Blood Cancer. 2020;67:e28409. PubMed https://doi.org/10.1002/pbc.28409

21. Bouffet E, Challinor J, Sullivan M, Biondi A, Rodriguez-Galindo C, Pritchard-Jones K. Early advice on managing children with cancer during the COVID-19 pandemic and a call for sharing experiences. Pediatr Blood Cancer. 2020;67:e28327. PubMed https://doi.org/10.1002/pbc.28327

22. Ding YY, Ramakrishna S, Long AH, Phillips CA, Montiel-Esparza R, Diorio CJ, et al. Delayed cancer diagnoses and high mortality in children during the COVID-19 pandemic. Pediatr Blood Cancer. 2020 Jun 6 [Epub ahead of print]. PubMed https://doi.org/10.1002/pbc.28427

23. Clarke JN, Fletcher P. Communication issues faced by parents who have a child diagnosed with cancer. J Pediatr Oncol Nurs. 2003;20:175–91. PubMed https://doi.org/10.1177/1043454203254040

24. Auletta JJ, Adamson PC, Agin JE, Kearns P, Kennedy S, Kieran MW, et al. Pediatric cancer research: surviving COVID-19. Pediatr Blood Cancer. 2020;67:e28435. PubMed https://doi.org/10.1002/pbc.28435

Appendix Table 1. Descriptive statistics of the COVID-19 misinformation items in this study

Misinformation item	Non-pediatric cancer parent, mean	Pediatric cancer parent, mean	*t*	p value
It is unsafe to receive a letter or package from China.	2.91	3.46	–5.59	<0.001
Pets at home can spread COVID-19.	2.79	3.12	–3.38	0.001
Vaccines against pneumonia can protect against COVID-19.	2.26	3.02	–8.15	<0.001
Regularly rinsing your nose with saline can help prevent infection with the COVID-19 virus.	2.59	3.26	–7.21	<0.001
Eating garlic can help prevent infection with the COVID-19 virus.	2.05	3.07	–10.91	<0.001
COVID-19 only affects older persons.	1.69	2.59	–8.99	<0.001
Antibiotics are effective in preventing and treating COVID-19.	2.19	3.13	–10.04	<0.001
The COVID-19 virus is just a mutated form of the common cold.	2.28	2.98	–7.25	<0.001
The future COVID-19 vaccine will contain a microchip.	2.27	2.94	–6.99	<0.001
A vaccine for COVID-19 already exists.	2.15	2.96	–8.41	<0.001
Colloidal silver products can help prevent or protect against COVID-19.	2.35	3.08	–8.23	<0.001
Gargling with or swallowing bleach will help get rid of COVID-19.	1.56	2.77	–12.14	<0.001
COVID-19 is less deadly than the flu.	2.31	2.93	–6.02	<0.001
Drinking a sip of water every 15 min can prevent COVID-19.	2.00	2.93	–9.54	<0.001
Vitamin C supplements will stop you from catching COVID-19.	2.20	3.10	–9.37	<0.001
Essential oils will protect you from COVID-19.	1.92	2.93	–10.58	<0.001
The COVID-19 virus cannot be transmitted in hot and humid weather.	2.25	3.05	–8.34	<0.001

Appendix Table 2. Descriptive statistics of study covariates by parent group

Covariate	Category	Non-pediatric cancer parent, n (%)	Pediatric cancer parent, n (%)	χ^2/ *t*	p value
Gender	Female	282 (32.9%)	122 (38.7%)	58.70	<0.001
	Male	138 (67.1%)	193 (61.3%)		
Age, y, mean (SD)		42.07 (11.53)	36.49 (7.90)	7.31	<0.001
Race/ethnicity	White	368 (87.6%)	249 (79.0%)	9.81	0.002
	Nonwhite	52 (12.4%)	66 (21.0%)		
Education	Bachelor’s degree or higher	211 (50.2%)	256 (81.3%)	74.82	<0.001
	Less than bachelor’s degree	209 (49.8%)	59 (18.7%)		
COVID-19 stress, mean (SD)*		3.13 (1.26)	3.70 (1.11)	6.50	<0.001

*Measured on a 6-point Likert scale, from “not at all stressed” (0) to “extremely stressed” (5).

Appendix Table 3. Results of logistic regression for each misinformation item*

Misinformation item		OR	95% CI	p value
Unsafe to receive package from China	Male	1.29	0.93–1.79	0.126
	Age	0.99	0.97–1.01	0.237
	Nonwhite	1.02	0.67–1.55	0.931
	College degree	0.97	0.68–1.38	0.880
	COVID-19 stress	1.26	1.11–1.44	0.000
	Pediatric cancer parent	2.22	1.56–3.15	0.000
Pets at home can spread COVID-19	Male	1.25	0.90–1.73	0.185
	Age	0.99	0.98–1.01	0.262
	Nonwhite	0.84	0.54–1.29	0.434
	College degree	0.93	0.66–1.33	0.700
	COVID-19 stress	1.28	1.12–1.46	0.000
	Pediatric cancer parent	1.45	1.02–2.07	0.040
Pneumonia vaccines can protect	Male	1.87	1.26–2.78	0.002
	Age	0.99	0.97–1.01	0.349
	Nonwhite	0.95	0.57–1.55	0.831
	College degree	0.82	0.52–1.28	0.381
	COVID-19 stress	1.31	1.11–1.55	0.001
	Pediatric cancer parent	4.23	2.75–6.63	0.000

Misinformation item		OR	95% CI	p value
Rinsing nose with saline can prevent	Male	1.32	0.93–1.88	0.120
	Age	0.98	0.97–1.00	0.100
	Nonwhite	1.01	0.63–1.59	0.972
	College degree	1.80	1.21–2.70	0.004
	COVID-19 stress	1.34	1.16–1.55	0.000
	Pediatric cancer parent	2.39	1.65–3.48	0.000
Eating garlic can help prevent	Male	1.70	1.15–2.52	0.007
	Age	0.99	0.97–1.01	0.547
	Nonwhite	0.83	0.49–1.38	0.479
	College degree	1.68	1.06–2.70	0.030
	COVID-19 stress	1.45	1.23–1.72	0.000
	Pediatric cancer parent	3.83	2.51–5.92	0.000
Affects only older persons	Male	1.88	1.25–2.83	0.002
	Age	0.98	0.96–1.00	0.095
	Nonwhite	0.92	0.53–1.54	0.745
	College degree	1.66	1.03–2.72	0.041
	COVID-19 stress	1.51	1.27–1.81	0.000
	Pediatric cancer parent	2.40	1.56–3.74	0.000
Antibiotics are effective in preventing/treating	Male	2.07	1.41–3.04	0.000
	Age	0.98	0.96–1.00	0.028
	Nonwhite	0.84	0.50–1.38	0.494
	College degree	1.87	1.19–2.98	0.007
	COVID-19 stress	1.40	1.19–1.65	0.000
	Pediatric cancer parent	3.36	2.24–5.08	0.000
COVID-19 is just mutated form of common cold	Male	1.87	1.30–2.70	0.001
	Age	0.98	0.96–1.00	0.054
	Nonwhite	0.82	0.50–1.31	0.419
	College degree	1.11	0.74–1.68	0.606
	COVID-19 stress	1.24	1.07–1.44	0.005
	Pediatric cancer parent	2.16	1.47–3.21	0.000
Vaccine will contain microchip	Male	1.73	1.18–2.54	0.005
	Age	1.00	0.98–1.02	0.865
	Nonwhite	0.75	0.45–1.24	0.279
	College degree	1.09	0.71–1.69	0.694
	COVID-19 stress	1.36	1.16–1.60	0.000
	Pediatric cancer parent	2.96	1.96–4.52	0.000
Vaccine already exists	Male	1.74	1.20–2.54	0.004
	Age	0.98	0.96–1.00	0.081
	Nonwhite	0.57	0.34–0.94	0.032
	College degree	0.78	0.51–1.19	0.248
	COVID-19 stress	1.37	1.17–1.60	0.000
	Pediatric cancer parent	2.58	1.73–3.89	0.000
Colloidal silver helps prevent or protect	Male	1.82	1.22–2.73	0.003
	Age	0.97	0.95–1.00	0.021
	Nonwhite	0.54	0.30–0.92	0.029
	College degree	1.42	0.89–2.29	0.148
	COVID-19 stress	1.43	1.21–1.71	0.000
	Pediatric cancer parent	3.27	2.13–5.09	0.000
Gargling or swallowing bleach will help	Male	2.45	1.63–3.73	0.000
	Age	0.97	0.95–1.00	0.020
	Nonwhite	0.87	0.50–1.46	0.602
	College degree	1.68	1.03–2.79	0.042
	COVID-19 stress	1.47	1.23–1.76	0.000
	Pediatric cancer parent	3.37	2.17–5.32	0.000
COVID-19 is less deadly than flu	Male	1.33	0.93–1.89	0.116
	Age	0.99	0.97–1.01	0.181
	Nonwhite	0.63	0.39–1.01	0.060
	College degree	0.97	0.66–1.43	0.880
	COVID-19 stress	1.17	1.01–1.35	0.033
	Pediatric cancer parent	2.16	1.48–3.17	0.000
Sip of water every 15 min prevents	Male	2.14	1.47–3.14	0.000
	Age	0.97	0.94–0.99	0.001
	Nonwhite	0.67	0.39–1.10	0.122
	College degree	2.23	1.43–3.55	0.001
	COVID-19 stress	1.23	1.05–1.44	0.009
	Pediatric cancer parent	2.07	1.39–3.10	0.000
Vitamin C supplement helps prevent	Male	1.60	1.11–2.30	0.011
	Age	0.98	0.96–1.00	0.017
	Nonwhite	0.67	0.41–1.08	0.107

Misinformation item		OR	95% CI	p value
	College degree	1.39	0.92–2.10	0.121
	COVID-19 stress	1.34	1.16–1.56	0.000
	Pediatric cancer parent	2.70	1.84–3.97	0.000
Essential oils protect	Male	1.79	1.20–2.67	0.004
	Age	0.97	0.95–0.99	0.015
	Nonwhite	0.62	0.35–1.05	0.081
	College degree	1.67	1.04–2.72	0.035
	COVID-19 stress	1.46	1.23–1.74	0.000
	Pediatric cancer parent	3.22	2.10–4.99	0.000
No transmission in hot weather	Male	1.53	1.06–2.22	0.023
	Age	0.98	0.96–1.00	0.040
	Nonwhite	0.99	0.61–1.59	0.975
	College degree	1.50	0.99–2.31	0.059
	COVID-19 stress	1.27	1.10–1.49	0.002
	Pediatric cancer parent	2.84	1.92–4.22	0.000

*CI, confidence interval; OR, odds ratio.
